# Complications of Tubularized Incised Plate Urethroplasty and Spongioplasty Repair in Hypospadias

**DOI:** 10.7759/cureus.94834

**Published:** 2025-10-17

**Authors:** Waleed Anjum Qureshi, Umama Jelani, Hamid Fazeel, Shahid Khan, Malik Junaid, Noor Ul Islam, Muhammad Ishaq, Wasim Khan

**Affiliations:** 1 Plastic Surgery, Northwest General Hospital and Research Centre, Peshawar, PAK; 2 Orthopaedics Surgery, Northwest General Hospital and Research Centre, Peshawar, PAK

**Keywords:** hypospadias, meatal stenosis, snodgrass repair, tips, urethra-cutaneous fistula, urethral stenosis

## Abstract

Background

Hypospadias is a common congenital anomaly in males, typically presenting with a ventrally displaced urethral meatus, incomplete prepuce, and chordee. Among over 300 surgical techniques described, the tubularized incised plate (TIP) urethroplasty with spongioplasty (TIPS), also known as the Snodgrass repair, is widely preferred for distal hypospadias due to its functional and aesthetic outcomes.

Objective

This study aims to determine the short-term complications of TIPS repair in pediatric patients with distal hypospadias.

Methods

A cross-sectional study was conducted at the department of burns, plastic and reconstructive surgery, Northwest General Hospital & Research Center, Peshawar, Pakistan, from June 2022 to March 2023. A total of 113 male patients (aged 18 months to 12 years) with distal hypospadias and a urethral plate width greater than 8 mm were included; 110 completed follow-up. Patients with proximal hypospadias or severe chordee were excluded. Postoperative complications were assessed over a minimum follow-up of three months.

Results

Of 110 patients, eight (7.27%) developed complications. Urethrocutaneous fistula (UCF) was the most common, occurring in six patients (5.45%), followed by urethral stricture in two (1.8%). No cases of meatal stenosis or chordee recurrence were observed. A statistically significant association was found between complications and both fistula (p = 0.0001) and stricture (p = 0.002).

Conclusion

TIPS repair is a safe and effective surgical option for distal hypospadias, demonstrating low short-term complication rates. UCF remains the most prevalent postoperative issue. Further long-term, multicenter studies are warranted.

## Introduction

Hypospadias is a male congenital defect. The meatus is situated on the ventral aspect of the urethra rather than on its tip. It is accompanied by an incomplete hooded prepuce with or without ventral curvature called chordee. Hypospadias is related to the ventral curvature of the penis (chordee). Hypospadias is associated with functional and psychological challenges for both patients and their parents [1-3]. Hypospadias occurs in approximately one in every 125 male births. The incidence has doubled from 1970 to 1993. Although reports suggest that this increase reflects increased reporting of minor grades of hypospadias, increases in severe hypospadias were also noted. Some reports linked the increased rate of hypospadias in boys born prematurely and small for gestational age and boys with low birth weight [4].

The disorder is identified clinically and managed surgically to improve certain psychological and physical aspects of life, such as voiding in the standing position and ejaculation at the tip. Due to various factors, surgery has been riddled with complications all over the world [5]. Consequently, long-term complications, with an incidence rate of 30%, have been observed after hypospadias repair. Hypospadias repair has long-term complications and various risk factors associated with it, such as hypospadias location, hypospadias severity, surgical repair method, perioperative hormonal therapy, dressing technique, and type of suture [6].

The Snodgrass repair, also referred to as tubularized incised plate (TIP) urethroplasty with spongioplasty (TIPS), has become a widespread repair technique for the correction of both proximal and distal hypospadias. TIPS was popularized in 1994 by Dr. Warren Snodgrass. This technique has shown reliable and effective results with a positive impact on the patient’s psychological and physical aspects of life and an acceptable rate of complications. A prominent advantage of this repairing technique is the development of a vertical slit (neo-meatus) with a wide urethral caliber and smooth closure. The most common postoperative complications involve urethrocutaneous fistula (UCF), meatal stenosis, urethral diverticulum, and stricture [7-9].

The Snodgrass repair technique is considered to be an effective method despite complications that pose risks to patients’ quality of life. The TIP repair has become the most commonly used repair for both distal and midshaft hypospadias. This includes a primary tubularization of the urethral plate, with incision of the posterior wall of the plate, which allows it to hinge forward. This creates a lumen of greater diameter than would otherwise be possible, obviating the routine use of a flap or graft to bridge the gap. The incised plate is left to urothelial use over the next few days. It is clear that proximal repairs are associated with a greater incidence of complications. Older age at surgery and low surgical experience have also been associated with poorer outcomes. A study found that staged repairs were associated with higher complication rates and that high-volume centers had lower complication rates. With longer follow-up, it is apparent that late complications can occur, and thus, most advocate continued evaluation through puberty [10]. A study showed that the incidence of secondary surgical repair of hypospadias is underreported if follow-up is limited to less than six years [11]. Infection is a rare complication of hypospadias repair in the modern era. Skin preparation and perioperative antibiotics are generally used. Patients are often maintained on an antibiotic course until any stents are removed, though this has not clearly been shown to be beneficial. UCF is a major concern in hypospadias repair. The rate of fistula formation is generally less than 6% for most single-stage repairs but rises with the severity of hypospadias, approaching 40% with complex re-operative efforts. Fistulas rarely close spontaneously and are repaired by using a multilayered closure with local skin flaps six months after the initial repair. After repair, no fistulas have been reported. Meatal stenosis, or narrowing of the urethral meatus, can occur. A urethral stent prevents any problems initially, but a fine-spraying urinary stream that is associated with straining to void may necessitate evaluation and possible surgical revision of the distal urethra [12]. Grafting the dorsal TIP incision was first considered an option to fill the defect and ensure healing without stenosis. Using the foreskin to graft the incision may preclude circumcision for families that prefer a natural appearance, and the foreskin may not be available for megameatus intact prepuce variants diagnosed after circumcision, but G-TIP is otherwise an option for most patients with distal hypospadias [13]. The complications of the Snodgrass technique pose risks to patients’ quality of life; as very little literature work is found on this topic in our country, the results of this study will help us and other practitioners in understanding these complications and adopting effective treatment for the proper care of the patient in pre- and postoperative scenarios and open new doors for further research in this field.

## Materials and methods

Study design and setting

This was a descriptive cross-sectional study conducted at the department of burns, plastic and reconstructive surgery, Northwest General Hospital & Research Center, Peshawar, Pakistan. The study period extended from June 1, 2022, to March 31, 2023.

Study population

A total of 113 male patients, aged between 18 months and 12 years, diagnosed with distal hypospadias and having a urethral plate width greater than 8 mm, were included in the study. Patients with severe chordee, proximal hypospadias, previously scarred urethral plates, or absent urethral plates were excluded to ensure uniformity in surgical technique and outcome assessment.

Ethical considerations

Ethical approval was obtained from the institutional review board. Informed written consent was obtained from parents or legal guardians after clearly explaining the purpose, procedures, risks, and benefits of the study.

Data collection procedure

Demographic information, such as age, area of residence, and socioeconomic status, was recorded. A thorough medical history and physical examination were performed preoperatively. All surgeries were conducted under general anesthesia with caudal analgesia. The operative procedure began with a midline glanular holding suture using 5/0 Prolene. The urethral plate and glans diameters were then measured.

A U-shaped incision was made around the urethral plate, extending dorsally across the penis. Penile degloving was performed from the dorsal to the ventral side. After complete degloving, the degree of curvature was reassessed. A midline incision was made in the urethral plate using fine scissors to widen it, and the width was re-measured.

Follow-up and outcome assessment

Patients were followed up for a minimum of three months to observe short-term complications associated with TIPS, also known as the Snodgrass repair. Three patients were lost to follow-up, resulting in 110 patients being included in the final analysis. Postoperative complications were recorded as per predefined operational definitions.

Data analysis

Data were analyzed using IBM SPSS Statistics, version 23.0 (IBM Inc., Armonk, NY). Frequencies and percentages were calculated for categorical variables such as types of complications. Mean and standard deviation were calculated for continuous variables. A poststratification chi-square test was applied to determine the association of complications with various demographic and clinical factors, with a p-value of <0.05 considered statistically significant. Results were presented in the form of tables for clarity.

## Results

TIPS for distal hypospadias

Table 1 presents the age distribution of the 110 male patients who underwent TIPS repair for distal hypospadias. The majority of patients (65, 59.1%) belonged to the age group of six to 12 years, while 45 patients (40.9%) were between one and five years of age. The mean age of the study population was 6.46 ± 3.50 years, indicating that most patients presented for surgical correction during the later phase of early childhood.

**Table 1:** Age distribution of patients

Age group	Frequency	Percentage
1-5 years	45	40.90%
6-12 years	65	59.10%
Total	110	100.00%

Table 2 summarizes the overall frequency of postoperative complications following the TIPS procedure. Out of the 110 patients, eight (7.27%) developed complications, while 102 (92.73%) had no postoperative complications during the follow-up period. This indicates a relatively low complication rate, supporting the safety and reliability of the Snodgrass technique in selected patients.

**Table 2:** Frequency of overall complications following TIPS repair TIPS, tubularized incised plate urethroplasty with spongioplasty

Complications present	Frequency	Percentage
Yes	8	7.27%
No	102	92.73%
Total	110	100.00%

Table 3 highlights the frequency of UCF, the most commonly observed complication in this study. UCF was seen in six patients (5.45%), while the remaining 104 patients (94.55%) did not exhibit this complication. This aligns with global data identifying UCF as one of the most frequent adverse outcomes following hypospadias repair.

**Table 3:** Frequency of UCF UCF, urethrocutaneous fistula

UCF present	Frequency	Percentage
Yes	6	5.45%
No	104	94.55%
Total	110	100.00%

Table 4 presents the incidence of urethral stricture postoperatively. Urethral stricture was found in two patients (1.8%), while 108 patients (98.2%) remained free of this complication. Though less common than UCF, urethral stricture remains a significant issue due to its potential for long-term morbidity.

**Table 4:** Frequency of urethral stricture

Urethral stricture	Frequency	Percentage
Yes	2	1.80%
No	108	98.20%
Total	110	100.00%

Table 5 presents the stratification of complications with the presence of UCF. Among the eight patients who developed complications, six (75%) had UCF, while two (25%) had other complications. None of the 102 patients without complications developed UCF. A chi-square test of independence indicated a statistically significant association between overall complications and UCF occurrence (χ²(1, N = 110) = 85.71, p < 0.0001, φ = 0.88), suggesting a strong relationship.

**Table 5:** Stratification of complications with UCF UCF, urethrocutaneous fistula

Complication status	UCF present	UCF absent	Total	p-value
Yes	6	2	8	0.0001
No	0	102	102	
Total	6	104	110	

Table 6 examines the relationship between complications and meatal stenosis. There were no recorded cases of meatal stenosis in either group. The chi-square test yielded no statistical significance (χ²(1, N = 110) = 0.00, p = 1.00, φ = 0.00), confirming the absence of association.

**Table 6:** Stratification of complications with meatal stenosis

Complication status	Meatal stenosis present	Absent	Total	p-value
Yes	0	8	8	1.00
No	0	102	102	
Total	0	110	110	

Table 7 presents the stratification of complications by urethral stricture. Among eight patients with complications, two (25%) developed urethral strictures, while none of the 102 patients without complications had strictures. The chi-square test revealed a statistically significant association (χ²(1, N = 110) = 25.31, p = 0.002, φ = 0.48), indicating a moderate effect size.

**Table 7:** Stratification of complications with urethral stricture

Complication status	Urethral stricture present	Absent	Total	p-value
Yes	2	6	8	0.002
No	0	102	102	
Total	2	108	110	

## Discussion

In this study involving 110 patients with distal hypospadias who underwent TIPS repair, the overall complication rate was 7.27%. This rate falls within the lower spectrum of complication rates reported in recent literature, which ranges between 5% and 15% depending on the patient population and surgical technique employed [14,15]. The relatively low complication rate in our cohort likely reflects strict patient selection criteria, including the exclusion of patients with severe chordee and proximal hypospadias, which are well-known risk factors for higher complication rates. One study reported complication rates of about 6.2% in a similarly selected cohort, emphasizing the significance of patient selection and surgical expertise in reducing adverse outcomes [14].

UCF was the most frequent complication observed, with a rate of 5.45%. This aligns closely with recent studies that have identified UCF as the primary postoperative complication following the Snodgrass repair, with reported rates generally between 5% and 10% [16,17]. The incidence of fistula is influenced by numerous factors, including surgical technique, tissue coverage, and tension on the repair. A study found that the use of well-vascularized second-layer flaps such as dartos or tunica vaginalis significantly reduces fistula formation, emphasizing the importance of meticulous dissection and multilayer closure [17]. Our approach, which included careful penile degloving and spongioplasty, likely contributed to the relatively low fistula rate, consistent with previous findings that highlighted the role of Buck’s fascia in reinforcing the repair and minimizing fistula risk [18]. Nonetheless, fistula formation remains a challenging complication, particularly in complex cases, underscoring the need for ongoing refinement in surgical technique.

The incidence of urethral stricture in our study was 1.8%, which is at the lower end of the range reported in recent meta-analyses, where stricture rates vary from 2% to 10% after hypospadias repair [19]. Stricture formation is multifactorial but is closely linked to the characteristics of the urethral plate, surgical technique, and postoperative healing. A study found that a wider urethral plate (greater than 8 mm) significantly reduces the risk of stricture, corroborating our inclusion criteria that required a urethral plate width greater than 8 mm [20]. Furthermore, the midline incision technique used in our repairs to widen the urethral plate and tension-free suturing are critical steps known to decrease the risk of stricture, as found in a study highlighting the technical nuances that impact stricture development [21]. These findings suggest that careful preoperative assessment of the urethral plate and adherence to surgical principles can minimize this serious complication.

Interestingly, no cases of meatal stenosis or chordee recurrence were recorded in our cohort, which contrasts with some recent reports where meatal stenosis rates ranged from 3% to 6% following hypospadias repair [16]. This discrepancy may be explained by our surgical technique, which included meticulous glansplasty and careful intraoperative assessment of penile curvature, as well as the exclusion of patients with severe chordee, a known risk factor for curvature relapse [14]. The absence of chordee recurrence is encouraging and aligns with current recommendations that emphasize complete penile degloving and intraoperative correction of curvature as essential steps to prevent postoperative chordee [18,19]. However, it is important to consider that longer follow-up periods are often required to fully assess the incidence of late complications such as chordee relapse.

Overall, our findings reinforce that TIPS repair is an effective and safe surgical option for distal hypospadias when performed with appropriate patient selection and careful surgical technique. The low rates of major complications in this study are comparable to those reported in recent high-quality studies and meta-analyses [14-21]. However, the relatively short minimum follow-up of three months limits the detection of late complications, including delayed strictures, cosmetic dissatisfaction, or functional issues that may manifest over time. Additionally, this single-center study’s exclusion of more severe hypospadias variants limits the generalizability of the results to all hypospadias cases. Future multicenter prospective studies with longer follow-up and inclusion of a broader spectrum of hypospadias types are needed to better characterize complication profiles and optimize surgical outcomes.

## Conclusions

The findings of this study affirm that TIPS is a reliable and effective surgical technique for the correction of distal hypospadias, demonstrating a favorable safety profile. UCF emerged as the most commonly encountered postoperative complication, followed by urethral stricture, both showing a significant association with adverse surgical outcomes. No instances of meatal stenosis or chordee recurrence were observed during the follow-up period. These results underscore the importance of appropriate patient selection and precise surgical execution in achieving positive short-term outcomes. The findings align with existing literature and support the continued use of the Snodgrass repair in suitable cases. Nonetheless, longer-term, multicenter studies are recommended to assess delayed complications and enhance postoperative management protocols.
